# Premenstrual Syndrome and Comorbid Depression Among Medical Students in the Internship Stage: A Descriptive Study

**Published:** 2014

**Authors:** Seyed Saeed Sadr, Seyed Mehdi Samimi Ardestani, Katayoon Razjouyan, Mahboobeh Daneshvari, Ghazal Zahed

**Affiliations:** 1 Assistant Professor, Department of Psychiatry, Imam Hossein Medical Center, Behavior Sciences Research Center, Shahid Beheshti University of Medical Sciences, Tehran, Iran.; 2 Associate Professor, Department of Psychiatry, Imam Hosein Medical Center, Behavior Sciences Research Center, Shahid Beheshti University of Medical Sciences, Tehran, Iran.; 3 Assistant Professor, Department of Child and Adolescent Psychiatry, Imam Hosein Medical Center, Behavior Sciences Research Center, Shahid Beheshti University of Medical Sciences, Tehran, Iran.; 4 General Practitioner, Tehran, Iran.; 5 Assistant Professor, Department of Child and Adolescent Psychiatry, Mofid Medical Center, Behavior Sciences Research Center, Shahid Beheshti University of Medical Sciences, Tehran, Iran

**Keywords:** Depression, Medical Students, Premenstrual Dysphoric Disorder, Premenstrual Syndrome

## Abstract

**Objective:**

Premenstrual syndrome (PMS) is a cluster of physical and emotional changes that typically begins several days before the menstrual period that disappears quickly after menstruation. It seems that co-occurrence of depression increases the risk and severity of this syndrome. In this cross-sectional research, we evaluated an association between PMS and depression in medical students.

**Methods:**

A hundred female medical students of Shahid Beheshti University of Medical Sciences that were available assigned for research. They were divided into two groups after administration of demographic questionnaire and PMS questionnaire made by researchers based on Diagnostic and Statistical Manual of Mental Disorders, Fourth Edition-Technical Revision; group with or without PMS diagnosis. Then, they completed Beck’s Depression Inventory.

**Results:**

From 100 participants, 55% (n = 55) met the PMS criteria and 45% had no PMS. In the PMS group 30% (n = 17) had no depression; 38% (n = 21) had mild depression; 23% (n = 13) had moderate depression; and 7% (n = 4) had severe depression. In the group with no PMS 60% (n = 27) had no depression; 20% (n = 9) had mild depression; 17% (n = 8) had moderate depression; 2% (n = 1) had severe depression. The rate of depression was significantly higher in PMS group (p = 0.04).

**Conclusion:**

In this research, PMS had an elevated frequency in medical students. In students with PMS, rate of depression was higher than students without PMS.

## Introduction

Premenstrual syndrome (PMS) and the most severe form of it, premenstrual dysphoric disorder (PMDD) is a common problem in the reproductive age (1-3). It is characterized by physical and psychological symptoms that can result in significant impairments (4-6). The symptoms begin 1-2 weeks before the menstrual period (the luteal phase of the menstrual cycle) and subside rapidly after the onset of menstruation (7). Although the prevalence of full-blown PMDD varies among studies, it is estimated that 3-8% of women suffer from it (8-10), and about 30-50% of menstruating women have some PMS symptoms (7). Common disorders that may co-occur with PMS are major depression disorder, dysthymic disorder, bipolar disorder, panic disorder, generalized anxiety disorder, and hypercholesterolemia (7, 11-13). The management of PMDD/PMS has to include assessment and paying special attention to suicide, also this syndrome should keep in mind in regard to every woman who attempted or have suicidal ideation (14). Similar to most disorders in psychiatry, PMDD/PMS and comorbid depression have bilateral negative impacts on the severity of each other. It means that the severity of each depression and PMS can affect the presentation or the severity of the other (7), so recognizing coincident disorders and subsequent treatment seems to be effective in reducing morbidity.

In some studies, it has been shown that hormones and contraceptive drugs are effective for the treatment of PMS, especially in more severe forms (PMDD) (2, 15, 16). This indicates that hormonal imbalance has an important role in the pathophysiology of the syndrome. On the other hand, besides biologic (such as hormonal imbalance during the menstrual cycle) and temperamental factors (17-19), social factors (20) and work stresses may have a substantial role in producing the PMS/PMDD (18, 21, 22). Medical workers, including physicians and medical students are among high-stress employees (23-27). Therefore, it is predictable that depression and PMS have elevated frequencies in this population. In spite of various frequencies of PMS/PMDD in different studies, all surveys detected high rate of this syndrome among medical students (28-30).

The aim of this cross-sectional study was to determine the frequency of PMS as well as comorbid depression in Iranian medical students by internship period of medical education.

## Materials and Methods

It was a cross-sectional study, and participants were female medical students in the internship stage.

Participants were all female medical students of Shahid Beheshty University who were passing their internship period in medical education in 2011. The informed consent was obtained from them. Exclusion criteria were active non-psychiatric disorders, history of personality disorders, psychosis, polycystic ovarian disorder, endometriosis, pregnancy, and history of any mental disorder after childbirth. If any of participants have pre-menstrual symptoms at the time of research, date collection were postponed. Information about these medical and psychiatric diseases was collected by history taking from participant. Finally, 100 persons entered the survey by available sampling.

After explaining the procedure, three questionnaires were filled by researchers:

1. A demographic questionnaire which is contained personal information.

2. Checklist of PMS symptoms: It included 11 questions related to PMS symptoms according to Diagnostic and Statistical Manual of Mental Disorders, Fourth Edition-Technical Revision. The emphasis of the questionnaire has been on the symptoms appearing just 1 week before the menstrual and disappearance of them after the onset of menstruation. If at least five answers of 11 questions were yes, subjects were eligible for PMS.

Subjects were eligible for PMS if at least five answers of 11 questions had been positive.

Beck Depression Inventory-II: Including 21 multiple questions that are graded from 0 to 3 based on the severity of symptoms. The lowest score is zero and the maximum 63. Scores between 0-9, 10-16, 17-29, and 30-63 indicate minimal, mild, moderate, and severe depression, respectively. Chi-square and ANOVA were used for analyzing categorical and continuous variables, respectively. It made by utilizing SPSS for Windows (version 19, SPSS Inc., Chicago, IL, USA).

## Results

From 100 participants, 55% (n = 55) met the PMS criteria and 45% had no PMS. In the PMS group 30% (n = 17) had no depression; 38% (n = 21) had mild depression; 23% (n = 13) had moderate depression; and 7% (n = 4) had severe depression. In the group with no PMS 60% (n = 27) had no depression; 20% (n = 9) had mild depression; 17% (n = 8) had moderate depression; 2% (n = 1) had severe depression (Table 1). Any kind of depression was more common in PMS group than subjects without PMS group. These difference were significant (p = 0.04).

Twenty-seven subjects (27%) were married and 73 (73%) unmarried. About 48% of married people and 57% of single people fulfilled PMS criteria. The statistical difference between these two subject groups was not significant (p = 0. 40) (Table 1). There was no significant difference between depression in two groups (56% of the married subjects versus 54% of single subjects) (p = 0.69).

PMS was a discriminative factor in regard to depression severity that is, the degree of beck inventory was significantly higher in students with this syndrome than those without it (p = 0.04) (Table 2).

About 56% of the married and 54% of singles had some degree of depression (Table 3). Married and single students had not significant differences in regard to depression score (p = 0.69).

## Discussion

According to this study, the total frequency of PMS in medical students in the internship stage was 55%, which was similar to the Namavar et al. findings (21). Our finding was somehow different from Nigeria study (36%) (28). The Nigerian samples were medical students who were attending in stages prior to internship period, and they were not involved in the shift-work state arranged during the internship course. Difference may be due to starting day-night shift and increasing stress in internship period. In addition, Namavar et al.’s (21) and our study sample were Iranian medical students; although, the prevalence of PMS varies in cross-countries researches (31, 32), it is unclear whether the PMS is more prevalent in Iran than Nigeria and this epidemiologic variation can explain the difference.

In addition, our study sample was similar to Namavar’s group study according to culture. It is unclear whether the PMS is more common in some cultures compare to others or something else made that difference. Why depression and its severity are positively correlated to the PMS? It can be explained by some hypothesis: 1) Depressed persons may have reduced tolerance of physical and psychological discomfort and report more physical symptoms (including PMS). 2) It might be a simple comorbidity. It seems like many other psychiatric disorders; by increasing the severity of depression, the probability of comorbidity will rise. 3) PMS could be a type of mood dysregulation due to hormonal changes during the menstrual cycle. It means depression may begin or, if present, become more severe in vulnerable women when hormonal balance disturbs (17, 33, 34).

Our study also showed psychiatrists must be more aware that PMS may not be a lonely syndrome. If clinicians do not pay attention to comorbid disorder, it could lead to incomplete treatment and continues morbidity; hence, careful evaluation of other psychiatric disorders in these people can be highly recommended. 

**Table 1:** Rate of premenstrual syndrome (PMS) regarding to marital status and PMS in medical students

**Marital status**	**With PMS** †	**Without PMS** †	**Total**
**Married**	13	14	027
**Single**	42	31	073
**Total**	55	45	100

† Premenstrual syndrome

**Table 2:** Severity of depression in students with premenstrual syndrome and those without

**Beck classification**	**With PMS** †	**Without PMS** †	**Total**
**Minimal (0-9)**	17	27	044
**Mild (10-16)**	21	09	030
**Moderate (17-29)**	13	08	021
**Severe (30-63)**	04	01	005
**Total**	55	45	100

† Premenstrual syndrome

**Table 3:** Depression severity in regarding to marital status

**Marital status**	**With depression**	**Without depression**	**Total**
**Married**	16	11	027
**Single**	40	33	073
**Total**	56	44	100

In our study, the effect of marital status on PMS was not significant. To the best of our knowledge, marital state is not one of considerable social factors in this syndrome and our findings were consistent with previous studies. As a different point of view, although marital status can affect females’ lifestyle and stresses, it’s not powerful enough to change premenstrual state. The high prevalence of PMS/PMDD among medical students in their stressful stage of the educational program should be a warning sign for policy makers of medical education. They should be sensitive to symptoms and detect it as soon as possible. It can reduce the burden of a potentially troublesome health problem this group.

It is clear, depression and PMS affect each other that is, depression increases the severity of PMS and having PMS increases the probability of concurrent depression. Nevertheless, depression and PMS can work separately. For instance, sometimes antidepressant drugs are effective for relieving symptoms of PMS independent to their antidepressant properties (12). Hence, PMS and depression should be concerned as separate components, which need relatively independent, but interactive managements.

Relation between bipolar disorder and PMS has been proposed (33-35). Also, depression in the context of bipolarity is more severe in compare to unipolar depression (36). Our study also showed that PMS is more observed in severe form of depression. Hence, it can be considered as a caution that every time a physician visits a patient with PMS and coexists depression (especially severe forms) have to remember the probability of bipolar disorders. There is no need to emphasize that correct finding of bipolarity instead of wrong diagnosing of unipolar depression is critical because these two types of mood disorders have different treatments in spite of overlapping symptoms that is, prescribing antidepressants can be at least counterproductive (if not contraindicated) for some of the bipolar patients.

Some other variables have been shown to be associated to PMS phenomenon in medical students. These factors are rural residency, lower age at menarche, regularity of menses and family history (37). Some factors such as rural residency are social parameters and point to possible stressors. Similar to many other psychosocial factors, habitancy location probably is a general stressor that can aggravate the PMS. On the other hand, we can find the trace of genetic issues in producing dysphoria before menstruation. It is not clear that which types of etiologies (environmental or genetic) are more powerful for producing PMS.

***Limitations***

In this study, we did not asses all variables possible related to PMS, and it’s severity because there is no consensus on these factors. 

## Authors' contributions

SSS conceived and designed the evaluation, and participated to draft preparation. SMSA prepared the draft and the final version of the manuscript. KR revised the manuscript and helped to revise the manuscript. MD collected the clinical data, interpreted them and analyzed the clinical and statistical data. GZ helped to analysis of data and revised the manuscript. All authors read and approved the final manuscript of course.

## Notes

**Declaration of interest:** None.
